# Ion Torrent-based transcriptional assessment of a *Corynebacterium pseudotuberculosis* equi strain reveals denaturing high-performance liquid chromatography a promising rRNA depletion method

**DOI:** 10.1111/1751-7915.12020

**Published:** 2013-01-15

**Authors:** Thiago L P Castro, Nubia Seyffert, Rommel T J Ramos, Silvanira Barbosa, Rodrigo D O Carvalho, Anne Cybelle Pinto, Adriana Ribeiro Carneiro, Wanderson Marques Silva, Luis G C Pacheco, Christopher Downson, Maria P C Schneider, Anderson Miyoshi, Vasco Azevedo, Artur Silva

**Affiliations:** 1 Institute of Biological Sciences, Universidade Federal de Minas GeraisBelo Horizonte, Brazil; 2 Genome and Proteome Network of the State of Pará, Universidade Federal do ParáBrazil; 3 Institute of Health Sciences, Universidade Federal da BahiaSalvador, Brazil; 4 School of Life Sciences, University of WarwickCoventry, UK

## Abstract

*Corynebacterium pseudotuberculosis* equi is a Gram-positive pathogenic bacterium which affects a variety of hosts. Besides the great economic losses it causes to horse-breeders, this organism is also known to be an important infectious agent to cattle and buffaloes. As an outcome of the efforts in characterizing the molecular basis of its virulence, several complete genome sequences were made available in recent years, enabling the large-scale assessment of genes throughout distinct isolates. Meanwhile, the RNA-seq stood out as the technology of choice for comprehensive transcriptome studies, which may bring valuable information regarding active genomic regions, despite of the still impeditive associated costs. In an attempt to increase the use of generated reads per instrument run, by effectively eliminating unwanted rRNAs from total RNA samples without relying on any commercially available kits, we applied denaturing high-performance liquid chromatography (DHPLC) as an alternative method to assess the transcriptional profile of *C. pseudotuberculosis*. We have found that the DHPLC depletion method, allied to Ion Torrent sequencing, allows mapping of transcripts in a comprehensive way and identifying novel transcripts when a *de novo* approach is used. These data encourage us to use DHPLC in future transcriptional evaluations in *C. pseudotuberculosis*.

## Introduction

*Corynebacterium pseudotuberculosis* is a Gram-positive and facultative intracellular bacterium that does not sporulate, has fimbriae, and displays pleomorphic cells which vary from coccoid to filamentary rods, measuring up to 0.6 μm per 3 μm in size (Jones and Collins, 1986; Baird and Fontaine, 2007). Two biovars have been described for *C. pseudotuberculosis*, ovis and equi; they are mainly distinguished by their ability to reduce nitrate and tropism for infecting particular kinds of animals (Biberstein *et al*., 1971).

Caprine, ovine, equine and bovine are among the most prominent hosts, to whom the infection by *C. pseudotuberculosis* can cause distinct clinical signals. The nitrate-negative biovar ovis, the most studied of *C. pseudotuberculosis*, causes caseous lymphadenitis in sheep and goats, a chronic disease characterized by the abscessation of external and internal lymphnodes. On the other hand, the biovar equi commonly tests positive for nitrate reduction, and is associated to distinct diseases in a variety of hosts, including ulcerative lymphangitis in horses and contagious acne in cattle (Paton *et al*., 1994; Braverman *et al*., 1999; Spier *et al*., 2004). *Corynebacterium pseudotuberculosis* biovar equi is also the causative agent of oedematous skin disease in buffaloes, characterized by superficial swellings which culminate in the formation of abscesses and release of serous bloody exudates (Barakat *et al*., 1984).

Recent efforts in elucidating the molecular features associated to host tropism and virulence have led to the acquisition of whole-genome sequences of six different strains of *C. pseudotuberculosis* equi, all of them made public in the National Center for Biotechnology Information databases (NCBI). However, additional studies comparing such sequenced strains are necessary, similarly to what is already being done with biovar ovis specimens. Two distinct studies have lately benefited from genomic data to compare traits among the strains 1002 and C231, isolated from goat and sheep respectively. Ruiz and colleagues (2001) revealed that C231 lacks genes involved in gluconeogenesis and triacylglycerol degradation, while these pathways are likely to be present in 1002. Still, the availability of annotated features, combined to LC-MS^E^ proteomics, has allowed Pacheco and colleagues (2011) to accurately identify 49 proteins which are exported uniquely by one of these two strains.

The assessment of differences between intraspecies genomes is challenging due to their strong evolutionary links, but this process may be largely facilitated by associating high-quality genomic information with comprehensive transcriptomic data. In this context, the massively parallel RNA-sequencing technologies (so-called RNA-seq) stand out providing the ultimate strategy for validating regions in the genome which are predicted to be transcribed, as well as estimating their transcriptional levels. By performing RNA-seq of an organism, it is also possible to identify operon structures and set new coding sequences (CDS), including untranslated regulatory elements (ncRNAs), what allows a more precise annotation of features present in the genome (Sorek and Cossart, 2010). The first RNA-seq work involving *C. pseudotuberculosis* was published recently by Pinto and colleagues (2012). The authors describe a set of genes from the 1002 strain which are co-regulated in response to adverse environmental conditions, such as high temperature and elevated osmolarity in the growth medium.

Despite being of great value for structural genomics, RNA-seq experiments are limited by the large quantity of reads which correspond to ribosomal RNA sequences. This is explained by the fact that more than 80% of the overall synthesized RNA in bacteria and eukatyotes is ribosomal (Westermann *et al*., 2012). Therefore, it is usual to submit total RNA to rRNA subtraction prior to the cDNA library construction, using commercially available kits based on rRNA exonuclease digestion or hybridization with specific probes. In the specific case of eukaryotic transcriptomes, it is also possible to enrich mRNA molecules with poly(A) selection (Martin and Wang, 2011). Although the effectiveness of these methods can be questioned, due to the presence of residual rRNAs in sequencing samples and additional costs involved, none remarkable published work regarding RNA-seq analysis in prokaryotes have ignored the depletion step (van Vliet, 2010).

Considering the current demand on RNA-seq experiments for *C. pseudotuberculosis* and other related bacteria, and the desire to improve the rates of mRNA sequences compared with those obtained with a commercially available kit, we decided to test denaturing high-performance liquid chromatography (DHPLC) as an alternative method for rRNA depletion. This chromatographic method allows the separation of nucleic acid molecules according to their hydrophobic characters, relying on a nonporous alkylated poly(styrene divinylbenzene) bead matrix as stationary phase. (Azarani and Hecker, 2000). In spite of being usually applied to mutational analysis and gene mapping purposes, the DHPLC could fit our needs since fractional recovery of initially loaded samples for downstream applications is possible, besides the fact that differences between rRNAs and mRNAs are visualized in the UV (260 nm) absorption-based chromatograms. In order to evaluate the outcomes brought about by DHPLC treatment, we have chosen the Ion Torrent Personal Genome Machine (PGM™) for cDNA library sequencing, once it is recognized as providing satisfactory read lengths in a time- and cost-effective manner (Meldrum *et al*., 2011; Jünemann *et al*., 2012). Therefore, by assessing the transcriptional activity of a *C. pseudotuberculosis* equi strain, we report a novel strategy combining DHPLC rRNA depletion and high-throughput sequencing, which could be employed in additional works on this species, apart from potentially extended to other model organisms.

## Results and discussion

### DHPLC depletion of rRNA and qRT-PCR validation

**Figure 1:**
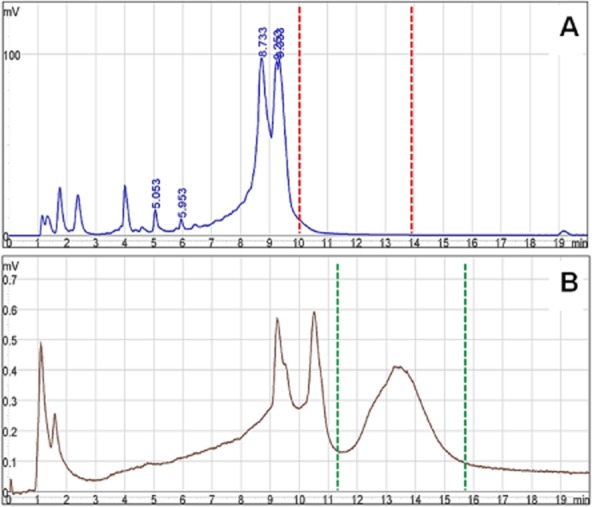
Chromatograms of a RNA sample before and after DHPLC fractionation for rRNA removal.A. Total RNA sample chromatographic profile. The two highest spikes correspond to ribosomal RNAs, and the red dashed lines delimitate the interval of run corresponding to the collected sample (mRNA) for downstream applications.B. Chromatographic profile of sample submitted to depletion with DHPLC. The green dashed lines virtually represent the previously selected area for sample collection. The proportion between the selected peak and the rRNA-related spikes appears to be more balanced in this case.

All extracted total RNA samples from *C. pseudotuberculosis* 31 showed similar chromatogram profiles after DHPLC analysis, such as the one illustrated in Fig. 1A. This is an important result for us, once in order to standardize the DHPLC rRNA depletion methodology we had to set a reliable interval representing our fraction of interest. Taking into account the WAVE® DHPLC System manufacturer's recommendations (Transgenomic) and the separation profiles generated for *C. pseudotuberculosis*, the retention period from 10 up to 14 min constituted the fraction to be collected for further evaluations of depletion in this work. The collected material was reanalysed using DHPLC, and a prominent peak likely to be formed by mRNA molecules came out in the chromatographic profile (Fig. 1B).

**Figure 2:**
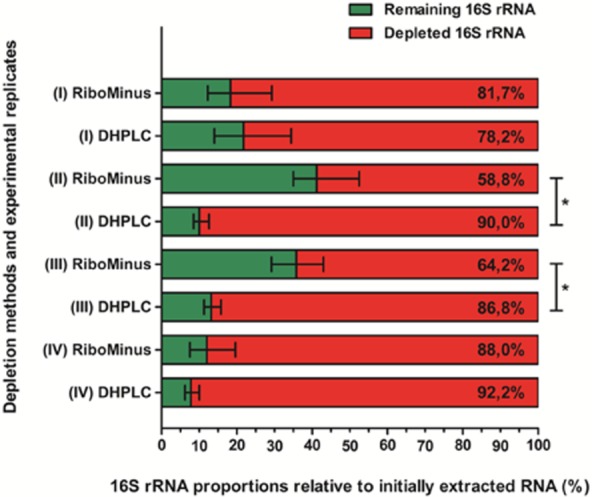
Percentages of 16S rRNA depletion for each of four independently prepared biological replicates, in relation to the *sigA* gene mRNA levels. DHPLC and RiboMinus treatments were performed in a parallel way for each replicate. The asterisks indicate the cases where the levels of depletion observed for DHPLC were significantly different from those seen for RiboMinus, according to the software REST 2009.

Quantitative reverse transcription PCR assays (qRT-PCR) showed partial removal of 16S rRNA after DHPLC fractioning, as occurred with samples treated in a parallel way using the commercial depletion kit RiboMinus (Chen and Duan, 2011) (Fig. 2). The rates of depletion with DHPLC ranged from 78.2% up to 92.2% in relation to the initial rRNA levels observed in non-treated total RNA samples. For the RiboMinus treatment, the rates ranged from 58.8% up to 88%, with a greater experimental variation in depletion efficiency when compared with what was seen for DHPLC. This leads us to suggest that the DHPLC fractionation method is probably less sensitive to minor inter-sample variations than the hybridization subtraction-based RiboMinus kit. However, it was not possible to state DHPLC as the most efficient depletion method, since it significantly demonstrated better performance in only two out of four independent experiments. In this context, it is prudent to consider that for our model organism, DHPLC provides at least the same depletion levels of a well-established and broadly trusted commercially available kit.

### Reference-based transcriptional assessment

The transcriptome of *C. pseudotuberculosis* 31 was assessed based on the RiboMinus- and DHPLC-depleted RNA samples, both derived from the same extraction but independently converted into cDNA for Ion Torrent sequencing. For RiboMinus, we estimated 58.75% of all sequences with Phred quality score greater or equal to 15 as being related to rRNA-coding genes (198 366 out of 337 645 reads), while for DHPLC the proportion of rRNA-related sequences decreased to 45.08% of total with Phred 15 score (183 963 out of 408 082 reads). The reads which have mapped against the CDS from the reference genome but are not ribosomal totalize 53 679 and 74 029 for RiboMinus and DHPLC respectively. The average length of total mapped reads was 143.35 nucleotide bases for RiboMinus and 144.59 bases for DHPLC.

**Figure 3:**
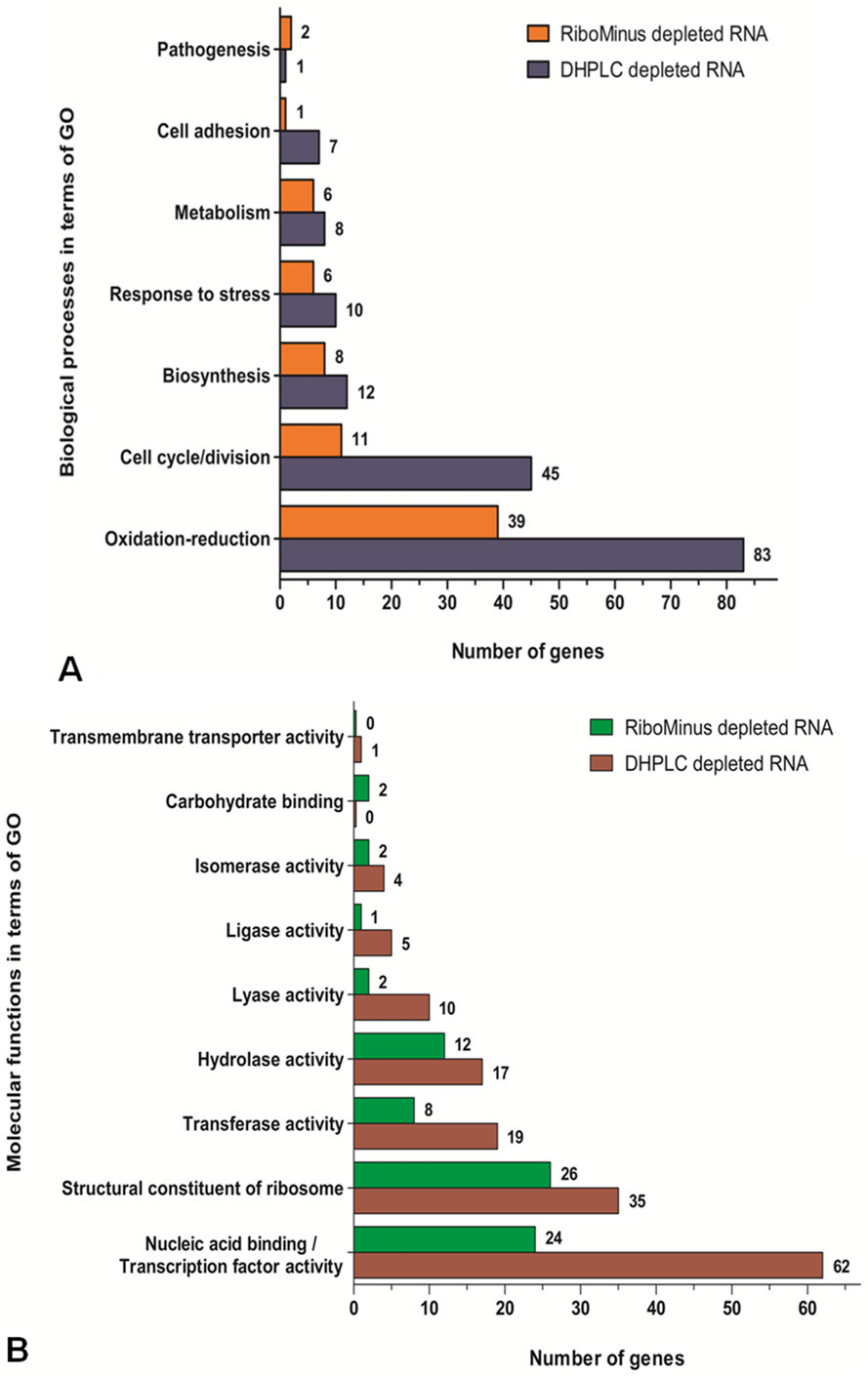
Classification of reads mapped against the CDS of *C. pseudotuberculosis* 31, according to terms of Gene Ontology.A. The identified transcripts were grouped by the biological processes to which their products are associated with. The horizontal bars indicate the amounts of grouped transcripts (genes) for each category, considering transcriptome results for both RiboMinus- and DHPLC-treated samples.B. In a similar way as before, the identified transcripts were sorted by their association with different classes of molecular functions.

For both transcriptomes, we investigated the representativity of biological processes and molecular functions attributed by Gene Ontology (GO) (Fig. 3). By comparing the samples, we noticed some similarity in the distribution of transcribed genes across the different classes considered, and this was expected since the same biological condition was addressed. However, the majority of classes presented a higher number of different transcripts in the case of DHPLC, what can be partially attributed to the larger number of non-ribosomal mapped reads acquired for the sample treated by this method. The process represented by the greatest number of transcripts was oxidation-reduction, whereas only a few transcribed elements were related to pathogenesis or cell adhesion, what is supported by the fact that the bacteria had been cultured without interacting with any hosts. Taking into account the functional classification, while transcripts for the class of structural constituents of ribosomes were the most abundant ones in the RiboMinus-treated sample, there was a greater representativity for the class of nucleic acid binding in the case of DHPLC depletion, including genes directly involved in transcriptional regulation.

Many of the identified transcripts were shared by both transcriptomes; among them we highlight the antigen 84, which is reported to be essential for *Mycobacterium smegmatis* polarization during cellular division, besides acting in the modulation of cell shape in pleiomorphic actinobacteria (Nguyen *et al*., 2007). Other interesting detected transcript codifies for a protein similar to the diphteria toxin (DT). The DT is described as the main virulence factor produced by *Corynebacterium diphtheriae*, and other proteins which are similar to DT in molecular weight and immunological structure have been detected in other pathogenic corynebacteria (Wong and Groman, 1984; Efstratiou *et al*., 1998). We can also mention the transcript for superoxide dismutase, known to be important for survival of *M. tuberculosis* inside activated macrophage cells (Piddington *et al*., 2001). Finally, we cite the transcripts for heat shock proteins (HSPs), which are involved in thermal stress adaptation and are also capable of inducing both humoral and cellular immune responses in the host (Pinho *et al*., 2009).

Among the transcripts uniquely identified following DHPLC fractionation we highlight those coding for a thioredoxin-related protein, engaged in oxidation-reduction processes; an urease accessory protein, which has been correlated to pathogenicity in corynebacteria; and the cell adhesion involved manganese ABC transporter substrate-binding protein and periplasmic zinc-binding protein TroA (Nolden *et al*., 2000; Hantke, 2005; Papp-Wallace and Maguire, 2006; Colbert *et al*., 2006). As uniquely identified in the RiboMinus-treated sample, we emphasize the transcripts linked to the transcriptional regulator sigma factor K and the detoxification-related mycothione glutathione reductase (Patel and Blanchard, 1999).

Analysis of the genome of *C. pseudotuberculosis* 31 using the Rfam software allowed us to predict eleven non-coding functional RNAs (ncRNAs), other than rRNAs and tRNAs, which belong to three distinct known families: riboswitches, ribozymes and small RNAs (Table 1). Many of these ncRNAs are codified by bacteria of the genus *Streptomyces* and *Clostridium* (Weinberg *et al*., 2010; Chen *et al*., 2011), but their functions are not yet well understood. Among the predicted features, eight ncRNAs were confirmed to be transcribed, of which the tmRNA appeared as the most represented one in both RiboMinus- and DHPLC-related transcriptomes. The *Bacillus subtilis* tmRNA is required for growth at high temperature condition, beyond under cadmium and ethanol *in vitro* exposure (Muto *et al*., 2000). Other ncRNA relatively known and whose transcript was detected only in the RiboMinus-treated sample is the 6C.1, which plays a role in dormancy in *Streptomyces coelicolor* (Swiercz *et al*., 2008). In conclusion, although it may seem that the RiboMinus performed slightly better than DHPLC in maintaining ncRNA molecules in the pool of RNAs subjected to sequencing, further evaluations are necessary to better compare the efficiency of the two depletion methods on this issue. We also speculate that alternative fractions following the DHPLC treatment can be tested on their content of ncRNAs or any other specific group of RNAs.

**Table 1:** Evaluation of ncRNAs present in *C. pseudotuberculosis* 31 by combining genomic with transcriptomic data

				Corresponding read counts (RNA-seq)	
ncRNAs predicted in the genome	Corresponding strand	Rfam bits score	Genomic stance^a^ (bp)	RiboMinus-treated sample	DHPLC-treated sample
tmRNA.1	+	136.29	605 717–606 098	1497	444
RNaseP_bact_a.1	−	250.04	1 601 151–1 601 563	16	13
cspA.1	+	55.15	231 046–231 384	12	5
msiK.1	−	54.15	398 399–398 458	0	1
Bacteria_small_SRP.1	+	55.35	149 269–149 361	0	1
mraW.1	−	48.99	1 528 518–1 528 622	1	0
6C.1	+	32.18	223 511–223 577	1	0
Cobalamin.2	+	72.55	724 215–724 451	2	0
Cobalamin.1	+	74.77	924 216–924 412	0	0
TPP.1	+	57.38	793 275–793 405	0	0
yybP-ykoY.1	−	40.90	1 362 468–1 362 614	0	0

a In reference to the whole-genome sequence of *C. pseudotuberculosis* 31 (NC_017730.1).

### *De novo* strategy for new transcripts screening

One key possibility arising from transcriptome assessments is the identification of non-predicted CDS in the genome. Aiming to evaluate how our RiboMinus- and DHPLC-depleted RNA-sequencing data perform to allow the detection of new genes in *C. pseudotuberculosis* 31, we assembled all reads that had not mapped against the CDS from the reference genome into contigs (Table 2). Despite observing a higher number of non-mapped reads for the DHPLC sample, and a higher total of assembled contigs in this case as well, the removal of redundant sequences evidenced a larger amount of different contigs for RiboMinus (3836 against 1247 for DHPLC) (Table 3). One explanation for such inversion resides in the fact that larger contigs could be generated for DHPLC, what is in accordance with its greater N50 value (455 against 399 for RiboMinus).

**Table 2:** Statistics concerning the use of Oases software in the de *novo* approach

rRNA depletion method	Non-mapped reads used^a^	Assembly round	Total of contigs assembled	N50 of the contigs	Total base pairs assembled
RiboMinus	85 600	MergeK25	674	282	172 401
		MergeK27	1 225	283	318 679
		MergeK29	2 358	288	623 789
		MergeK31	4 595	291	1 229 563
		MergeK33	9 032	296	2 454 627
		MergeK35	17 822	310	5 195 511
DHPLC	150 090	MergeK25	1 681	429	564 199
		MergeK27	2 740	441	949 402
		MergeK29	4 799	445	1 706 112
		MergeK31	8 834	447	3 200 016
		MergeK33	16 948	450	6 210 773
		MergeK35	32 659	456	12 134 747

a Reads which did not match against CDS from *C. pseudotuberculosis* 31 (query coverage < 60% and similarity < 70%).

**Table 3:** Statistics for the *de novo* approach after removal of redundant contigs using the Simplifier software

	rRNA depletion method	
Non-redundant contigs (transcripts)	RiboMinus	DHPLC
Total number	3 836	1 247
Total base pairs	1 392 183	449 947
N50	399	455
Longer	1 712	2 237
Smaller	103	100
With hits^a^	778 (407 620 bp)	760 (295 852 bp)
Without hits^a^	3 058 (984 563 bp)	487 (154 095 bp)

a Alignments performed against CDS of *C. psedutoberculosis* 31. Were considered negligible alignments < 30 bp with *e*-value > 1e-0.5).

Following redundancy removal, we found that 487 DHPLC assembled contigs and 3058 RiboMinus assembled ones presented no matches against CDS of *C. pseudotuberculosis* 31 (Table 3). These considerable amounts of dissimilar sequences could be explained by intrinsic sequencing errors, which have caused penalties to matches under the criteria adopted by us (alignments should be larger than or equal to 30 bp and present *e*-value lower than or equal to 1e-05). Furthermore, our reference genome underwent automatic annotation process based on previously annotated genomes from other *C. pseudotuberculosis* strains, so it is plausible to consider that a number of CDS may have not been annotated in strain 31.

To analyse the similarity of all unknown contigs to the non-redundant nucleotide collection from the NCBI, we decided to use more strict criteria: only matches of at least 100 bp and with *e*-value lower than or equal to 1e-05 were considered. In spite of sequencing errors, which may have hampered our analysis, we could identify 15 different genes that were not previously annotated in the *C. pseudotuberculosis* 31 genome, six out of them related to hypothetical proteins in other organisms and one of unknown function (Table 4). Among the newly identified features are various genes coding for surface-anchored proteins, which participate in the bacterial pilli assembly and belong in their majority to the Spa family. Predicted to be present in the *C. pseudotuberculosis* ovis FRC41 strain, these proteins were proved to mediate, in *C. diphteriae* and other pathogens, bacterial attachment and colonization of host tissues (Gaspar and Ton-That, 2006; Trost *et al*., 2010). The remaining new transcripts, exclusively identified for the DHPLC-depleted sample, codify for anaerobic dehydrogenase and subunits alpha and beta of nitrate reductase, enzyme which plays an important role in the nitrogen assimilation (Richardson *et al*., 2001).

**Table 4:** New gene products found among the *C. pseudotuberculosis* 31 assembled transcripts

Gene product	Number of matching contigs^a^	Average size of contigs (bp)	Matching strain/product	Depletion method used
Hypothetical protein	20	1510	*Propionibacterium acnes* HL053PA2 (HMPREF9565_01483 protein)	DHPLC/RiboMinus
Hypothetical protein	31	1235	*Propionibacterium acnes* HL074PA1 (HMPREF9574_00607 protein)	RiboMinus
Hypothetical protein	2	782	*Corynebacterium glutamicum* R (cgr 1268 protein)	DHPLC
Hypothetical protein	1	366	*Bacteroides capillosus* ATCC 29799 (BACCAP_03832 protein)	RiboMinus
Hypothetical protein	2	1040	*Collinsella aerofaciens* ATCC 25986 (COLAER_01671 protein)	RiboMinus
Conserved hypothetical protein	1	572	*Lactobacillus jensenii JV-V16*	RiboMinus
Unknown protein	1	344	*Streptococcus suis* 98HAH33	RiboMinus
*spaF* gene product	8	1732	*Corynebacterium pseudotuberculosis* FRC41	DHPLC
*spaE* gene product	6	880	*Corynebacterium pseudotuberculosis* FRC41	DHPLC
*spaY* gene product	3	642	*Corynebacterium pseudotuberculosis* FRC41	DHPLC
*spaC* gene product	1	958	*Corynebacterium ulcerans* 809	DHPLC
Surface-anchored fimbrial protein	8	958	*Corynebacterium ulcerans* BR-AD22	DHPLC
Anaerobic dehydrogenase	1	332	*Corynebacterium glutamicum ATCC 13032*	DHPLC
Nitrate reductase beta subunit	2	554	*Propionibacterium acnes* HL074PA1	DHPLC
nitrate reductase alpha subunit	4	651	*Corynebacterium diphtheriae* HC04	DHPLC

a Only matches ≥ 100 bp with *e*-value ≤ 1e-05 were considered.

With respect to these findings, we highlight that the DHPLC depletion method associated with Ion Torrent sequencing allowed us to successfully discover non-annotated genes in the genome of *C. pseudotuberculosis* 31, as occurred when RiboMinus was used. The next step to be taken is to thoroughly analyse and correct all annotation errors, collaborating with upcoming studies regarding this organism.

In case the differences observed between the RiboMinus- and DHPLC-treated samples are caused by peculiarities of each depletion procedure, additional transcriptome evaluations are necessary for indicating to what extent these could alter the set of transcripts to be sequenced. Recent improvements on Ion Torrent technology will certainly benefit such work, by providing larger reads in a more efficient high-throughput way, and therefore minimizing the adverse effects caused by errors in sequences. Still, we speculate that the DHPLC rRNA depletion method could also be applied to the RNA-seq analysis intended for differential expression purposes, representing a cost-effective alternative for those groups which have access to the required equipment, since the expenses with depletion kits surely overcome those associated with the reagents for chromatography. On the other hand, in cases where only restricted amounts of total RNA are available, the DHPLC method is not likely to provide sufficient material for downstream applications. In conclusion, this work outlines a new rRNA depletion strategy which will be strongly considered to be applied in our future works about the transcriptional activity of *C. pseudotuberculosis*.

## Experimental procedures

### Bacterial strain, growth condition and preparation of bacterial pellet

A *Corynebacterium pseudotuberculosis* equi strain, identified as 31, was previously isolated from an infected buffalo in Egypt and confirmed by biochemical and molecular methods. This strain was regularly cultivated in Brain Heart Infusion broth (BHI, HiMedia Laboratories) and stored at −80°C with 50% glycerol. For the transcriptional assessments, Tween 80 (Sigma-Aldrich) was added to the BHI medium at 0.05% to avoid clumping of cells in suspension. The inoculated bacteria were grown at 37°C, 140 r.p.m., until the exponential phase was reached (OD_600nm_ = 0.4). For each culture, 20 ml were centrifuged at 5000 r.p.m. for 5 min, and then 5 ml of RNAlater® stabilizing reagent (Ambion) were added prior to storing at −80°C.

### RNA extraction from *C. pseudotuberculosis* C31

Stabilized bacteria were thawed on ice and then pelleted at 5000 r.p.m. for 5 min. Then they were suspended in RLT buffer (Qiagen) and submitted to mechanical disruption, using glass beads with 0.1 mm of diameter (VK01, Berthold Technologies). Two homogenization cycles were performed at 6500 r.p.m., 30 s each, with interval of 30 s between them, in a Precellys®24 equipment (Bertin Technologies). The total RNA was purified using RNeasy Mini kit (Qiagen), according to the manufacturer's recommendations. Optional on-column DNase treatment was carried out with the RNase-Free DNase set (Qiagen). The RNA was eluted from the purification column using RNase-free water, yielding at least 140 μg according to the Qubit® Fluorometric Quantitation kit (Invitrogen). Additionally, the RNA integrity was checked by electrophoresis in 1% agarose gels, in an RNase-free environment (Sambrook and Russell, 2001). Total extracted RNAs were stored at −80°C until use.

### rRNA depletion

For the rRNA depletion through DHPLC, four subsamples of 20 μg of total RNA each, provenient from the same extraction, were separately injected into the WAVE® System 4500 equipment loaded with the RNASep™ Cartridge (Transgenomic®). This procedure was necessary to achieve, at the end of the process, depleted RNA in sufficient amount for subsequent assays (over than 200 ng). The chromatographic process was carried out at fully denaturing condition (75°C). The buffers A [0.1 M triethylammonium acetate (TEAA) at pH 7.0] and B [0.1 M TEAA (at pH 7.0) containing 25% acetonitrile] were used to elute samples at the flow rate of 0.9 ml min^−1^, under a gradient condition as described by Azarani and Hecker (2001): 38% to 40% B in 1 min, 40% to 60% B in 15 min, 60% to 66% B in 6 min, 66% to 70% B in 0.5 min, 70% to 100% B in 0.5 min, kept at 100% B for 1 min, 100% to 38% B in 1 min, kept at 38% B for 2 min. The fraction corresponding to rRNA-depleted sample was collected, and then the subsamples were co-precipitated with sodium acetate (3 M), ethanol and glycogen (20 mg ml^−1^). The RNA pellets were dried and resuspended with RNase-free water. The chromatograms were analysed using the Wave® System's Navigator™ software (Transgenomic®). For each procedure of rRNA depletion through the RiboMinus™ Transcriptome Isolation Kit (Invitrogen), 6 μg of total RNA were loaded and treated according to the manufacturer's recommendations. Each RiboMinus-depleted sample, as occurred for DHPLC, yielded over than 200 ng of RNA, according to the Qubit® Fluorometric Quantitation kit (Invitrogen). All depleted RNA samples were stored at −80°C prior to further manipulations.

### Quantitative RT-PCR analysis

Fifty nanograms of each RNA sample, submitted or not to rRNA depletion procedures, were converted into cDNA using the Affinity Script QPCR cDNA Synthesis Kit (Agilent Technologies), according to the manufacturer's protocol. Reverse transcription was conducted as the following steps: 5 min at 25°C, 5 min at 42°C, 15 min at 55°C, and 5 min at 95°C. For the real-time PCR assays, we employed two distinct sets of oligonucleotide primers. The first pair anneals to the 16S rRNA-coding region in the genome of *C. pseudotuberculosis*, promoting a 92 bp amplicon, and the second is complementary to the sigma factor A *locus* (*sigA* gene). Both pairs provided amplification efficiencies greater than 0.95 under the tested conditions. The reactions for relative quantification were prepared as follows: 7.5 μl Brilliant SYBR® Green qPCR reagent (Agilent Technologies), 0.375 μl ROX reference dye (50× diluted), 1 μl forward primer (2 pmol), 1 μl reverse primer (2 pmol), 2 μl template cDNA (10× diluted), and ultra-pure water to final 15 μl volume. All amplifications were performed using the Applied Biosystems 7900HT Real-Time PCR System supplied with the software SDS, version 2.4. Dissociation curves were performed as follows: 95°C for 15 s, 60°C for 15 s, and 95°C for 15 s. For each of the four experimental replicates, the generated cycle-threshold values were loaded into the REST 2009 software (Qiagen), and then the 16S rRNA levels were assessed in relation to the corresponding housekeeping *sigA* gene transcription levels, as described by Pfaffl and colleagues (2002). In general lines, the qRT-PCR assays were performed in accordance with Bustin and colleagues (2009). Following the relative quantification, the mean and standard error values calculated for the 16S rRNA were converted into percentages, which were considered for all comparisons between the depletion methods. The outcome data were plotted with the software GraphPad Prism, version 5.0 (GraphPad Software, San Diego, CA, USA).

### RNA-sequencing

For RNA-sequencing, we started from a single total RNA sample, which was subdivided and passed through two independent rRNA depletion procedures: one with the RiboMinus kit and the other based on the DHPLC method. The cDNA libraries were prepared using Ambion® RNA-Seq Library Construction Kit. Briefly, 200 ng of fresh depleted RNA were fragmented with RNase III. After clean up, specific adapters (Ion adaptor mix, Life Technologies) were hybridized with 100 ng of each sample. The reverse transcription was carried out following the manufacturer's recommendations, and the resulting cDNA samples were purified. Only molecules with more than 100 bp were selected using the Agencourt AMPure® XP reagent (Beckman). After this stage, the cDNA was amplified and then purified with PureLink® PCR Micro Kit (Invitrogen). Quantification of amplified DNA samples was performed with the Qubit® dsDNA HS Assay Kit (Invitrogen).

Subsequently, the IKA® Ultra-Turrax® Tube Drive system was applied for an emulsion PCR, performed with reagents from the Ion Template Reagents Kit and the Ion Template Preparation Kit (Life Technologies). In brief, the DNA fragments were bonded onto spheres (Ion Sphere™ particles) and then amplified. Following the amplification, the emulsion was broken with successive washes (Ion Template Solutions Kit, Life Technologies), and the spheres without template were removed using the Dynabeads® MyOne™ Streptavidin C1 Magnetic Beads (Invitrogen). The spheres-bonded amplified fragments were mixed with the Ion Torrent sequencing primers and the sequencing polymerase enzyme, using the Ion Sequencing Reagents Kit (Life Technologies). The resulting mixtures were loaded on the Ion 314™ Chip for sequencing with the Ion Torrent Personal Genome Machine™ (PGM) (Life Technologies). All procedures adopted are in agreement with what is recommended by the handbooks of Ion Sequencing Reagent Kit and Ion Xpress™ Template Kit (Life Technologies).

### Bioinformatic analysis

The Ion Torrent-generated reads were trimmed and filtered according to in-house bioinformatic scripts. Sequences presenting an average Phred quality score inferior to 15 were eliminated by the Quality Assessment Software (Ramos *et al*., 2011). The reference-based assessment relied on the genome of *C. pseudotuberculosis* 31, obtained from the NCBI (NC_017730.1); and alignments which covered at least 60% of query sequences and achieved 70% or more of similarity with annotated CDS were considered for our analysis. To categorize the gene products into biological processes and molecular functions, the transcripts were processed with the Blast2GO software (Conesa *et al*., 2005). The outcome data were plotted using GraphPad Prism.

With respect to the ncRNAs identification, genomes RefSeq of the CMNR group members were obtained from the NCBI, and then a local database was built for predictions with the GeneMark software (Lukashin and Borodovsky, 1998). Subsequently, the Rfam software (http://rfam.sanger.ac.uk/) was used for prediction of ncRNAs. The identification of transcribed ncRNAs was performed as described for the reference-based assessment.

For the *de novo* strategy, we used all non-mapped reads from the reference-based analysis on the Velvet assembler (Zerbino and Birney, 2008) combined with the Oases software (Schulz *et al*., 2012). In brief, initial assemblies with different k-mers (25, 27, 29, 31, 33 and 35) were generated with Velvet. Subsequently, the transcripts obtained in each assembly were added into a single file, which was then submitted to new assemblies with Oases (k-mers 25–35). All assembled contigs for each rRNA depletion method were combined into a single multifasta file, and then undesired redundancies were removed by the Simplifier software (Ramos *et al*., 2012). The resulting contigs were blasted against the CDS of *C. pseudotuberculosis* 31, and were considered negligible alignments of less than 30 bp and which presented *e-*value greater than 1e-05. The dissimilar transcripts were then selected and blasted against the NCBI non-redundant nucleotide collection, and were considered new genes the hits covering 100 bp or more of queries and presenting *e*-value lower than or equal to 1e-05.
